# Draft Genome Sequence of *Bacillus pumilus* CCMA-560, Isolated from an Oil-Contaminated Mangrove Swamp

**DOI:** 10.1128/genomeA.00707-13

**Published:** 2013-09-12

**Authors:** Daniela F. Domingos, Bruna M. Dellagnezze, Paul Greenfield, Luciana R. Reyes, Itamar S. Melo, David J. Midgley, Valéria M. Oliveira

**Affiliations:** Microbial Resources Division, Research Center for Chemistry, Biology and Agriculture (CPQBA), Campinas University (UNICAMP), Campinas, São Paulo, Brazila; CSIRO Mathematics, Informatics and Statistics, North Ryde, NSW, Australiab; Laboratory of Environmental Microbiology, EMBRAPA Environment, Jaguariúna, São Paulo, Brazilc; CSIRO Animal, Food, and Health Sciences, North Ryde, NSW, Australiad

## Abstract

*Bacillus pumilus* strain CCMA-560 was isolated from an oil-contaminated mangrove swamp and was shown to produce biosurfactants. The strain appears to be capable of degrading some plant cell wall-related compounds, including hemicelluose and pectin. Genes for biopolymer export and polysaccharide intercellular adhesin synthesis were also annotated.

## GENOME ANNOUNCEMENT

*Bacillus pumilus* strain CCMA-560 was isolated from sediment from an oil-contaminated mangrove swamp in Bertioga city, São Paulo, Brazil (23°53′41″S, 46°12′32″W). Contamination of the swamp occurred in 1983, when 35 million liters of crude oil was spilled into the environment (1). Three sediment cores (each 5 by 30 cm) were collected from the swamp, pooled, subjected to 14-day enrichment in modified Bushnell-Haas Broth amended with NaCl (0.2%) and crude oil (0.1%), and incubated at 30°C. After this period, the cells were plated by serial dilutions onto nutrient agar and incubated at 30°C for 24 to 48 h. The identification of one of the individual colonies based on 16S rRNA sequencing and phylogenetic analysis revealed that the bacterium was a strain of *Bacillus pumilus*. This strain, designated CCMA-560, was shown to produce at least one biosurfactant (data not shown) and may be of interest for various industrial applications, including oil recovery.

The genome of *B. pumilus* CCMA-560 was sequenced using Illumina HiSeq 2000, and the resultant 100-bp paired-end sequences were corrected using Blue (http://www.bioinformatics.csiro.au/blue) prior to being assembled using Velvet v.1.2.07 (*k* = 57) (2). The final draft genome assembly (contigs, >200 bp) had approximately 1,300× coverage and comprised 72 contigs with a total length of 3,844,811 bp. For this assembly, the mean and median contig lengths were 53,400 bp and 19,157 bp, respectively, with a mean G+C content of 43.74%. Forty-nine short contigs (each <200 bp and totaling 7,065 bp) were excluded from the GenBank submission; however, these sequences are available in the annotation at the Integrated Microbial Genomes Expert Review (IMG-ER) website (3) and mostly contain fragments of various rRNAs and tRNAs. All contigs were submitted to and annotated using the IMG-ER pipeline, which predicted a total of 4,018 protein-coding genes and 103 structural RNAs. Based on the the 16S rRNA gene, *B. pumilus* CCMA-560 shows 100% sequence identity with *B. pumilus* strains SARF-032 and ATCC 7061, both isolated from soil. The genome of *B. pumilus* CCMA-560 appears to contain genes for degrading plant cell wall-related compounds, including hemicelluloses and pectin, and simple sugars, including glucose, fructose, sucrose, and xylose. The draft genome of *B. pumilus* CCMA-560 also possesses genes involved in biopolymer export and polysaccharide intercellular adhesin synthesis, along with numerous genes involved in the quorum-sensing pathway. If these genes are expressed, they may be involved in biofilm formation and concomitant exopolysaccharide production (4). Further investigation of this genome may yield insights into its potential for biosurfactant production.

### Nucleotide sequence accession numbers.

This whole-genome shotgun project has been deposited at DDBJ/EMBL/GenBank under the accession no. AUYP00000000. The version described in this paper is version AUYP01000000.
